# Dysregulation of Transcription Factor Networks Unveils Different Pathways in Human Papillomavirus 16-Positive Squamous Cell Carcinoma and Adenocarcinoma of the Uterine Cervix

**DOI:** 10.3389/fonc.2021.626187

**Published:** 2021-05-19

**Authors:** Saloe Bispo, Ticiana D. J. Farias, Patricia Savio de Araujo-Souza, Ricardo Cintra, Hellen Geremias dos Santos, Natasha Andressa Nogueira Jorge, Mauro Antônio Alves Castro, Gabriel Wajnberg, Nicole de Miranda Scherer, Maria Luiza Nogueira Dias Genta, Jesus Paula Carvalho, Luisa Lina Villa, Laura Sichero, Fabio Passetti

**Affiliations:** ^1^Instituto Carlos Chagas, FIOCRUZ, Curitiba, Brazil; ^2^Department of Immunobiology, Biology Institute, Universidade Federal Fluminense (UFF), Niterói, Brazil; ^3^Laboratory of Immunogenetics and Histocompatibility, Department of Genetics, Universidade Federal do Paraná, Curitiba, Brazil; ^4^Department of Biochemistry, Instituto de Quimica, Universidade de São Paulo, São Paulo, Brazil; ^5^Instituto Oswaldo Cruz, FIOCRUZ, Rio de Janeiro, Brazil; ^6^Bioinformatics Group, Department of Computer Science, Interdisciplinary Center for Bioinformatics, Leipzig University, Leipzig, Germany; ^7^Bioinformatics and Systems Biology Laboratory, Federal University of Paraná, Curitiba, Brazil; ^8^Atlantic Cancer Research Institute, Moncton, NB, Canada; ^9^Laboratory of Bioinformatics and Computational Biology, Division of Experimental and Translational Research, Brazilian National Cancer Institute (INCA), Rio de Janeiro, Brazil; ^10^Discipline of Gynecology, Department of Obstetrics and Gynecology, Instituto do Cancer do Estado de São Paulo, Hospital das Clinicas da Faculdade de Medicina da Universidade de São Paulo, São Paulo, Brazil; ^11^Department of Radiology and Oncology, Faculdade de Medicina, Universidade de São Paulo, São Paulo, Brazil; ^12^Center for Translational Research in Oncology, Instituto do Cancer do Estado de São Paulo ICESP, Hospital das Clinicas da Faculdade de Medicina da Universidade de São Paulo FMUSP HC, São Paulo, Brazil

**Keywords:** cervical cancer, cervical adenocarcinoma, cervical squamous cell carcinoma, RNA-Seq, transcriptional regulatory network, lncRNA, miRNA sponge

## Abstract

Squamous cell carcinoma (SCC) and adenocarcinoma (ADC) are the most common histological types of cervical cancer (CC). The worse prognosis of ADC cases highlights the need for better molecular characterization regarding differences between these CC types. RNA-Seq analysis of seven SCC and three ADC human papillomavirus 16-positive samples and the comparison with public data from non-tumoral human papillomavirus-negative cervical tissue samples revealed pathways exclusive to each histological type, such as the epithelial maintenance in SCC and the maturity-onset diabetes of the young (MODY) pathway in ADC. The transcriptional regulatory network analysis of cervical SCC samples unveiled a set of six transcription factor (TF) genes with the potential to positively regulate long non-coding RNA genes *DSG1-AS1, CALML3-AS1, IGFL2-AS1*, and *TINCR*. Additional analysis revealed a set of MODY TFs regulated in the sequence predicted to be repressed by miR-96-5p or miR-28-3p in ADC. These microRNAs were previously described to target LINC02381, which was predicted to be positively regulated by two MODY TFs upregulated in cervical ADC. Therefore, we hypothesize *LINC02381* might act by decreasing the levels of miR-96-5p and miR-28-3p, promoting the MODY activation in cervical ADC. The novel TF networks here described should be explored for the development of more efficient diagnostic tools.

## Introduction

Cervical cancer (CC) is the fourth leading cause of death related to cancer in women worldwide (1) and the second cause of mortality in women aged 20–39 years. Higher mortality rates due to CC are observed within developing countries (2). The main risk factor for CC development is the persistent infection by high-risk human papillomavirus (HPV) (3). Squamous cell carcinoma (SCC) and adenocarcinoma (ADC) are the most common histological types of CC, comprising ~80% and 20% of the cases of all invasive CC cases, respectively (4). However, ADC cases are increasing, possibly due to differential failures in the effectiveness of screening programs for ADC detection as compared with SCC (5). Despite their epidemiological, histopathological, and prognostic differences, patients presenting cervical SCC or ADC receive identical treatments (4). Currently recommended treatments are successful for most cervical SCC lesions but frequently fail in treating cervical ADC (4, 5). This reinforces the need to identify molecular differences between cervical histological types. This knowledge is seminal for cancer biology understanding and to foster the development of novel ADC treatments.

Complex diseases, such as CC, can be evaluated by means of regulatory gene expression networks (6). This analysis identifies expression enrichment of specific transcription factors (TFs) as key regulators of various target genes, such as protein-coding, microRNAs (miRNAs), and long non-coding RNAs (lncRNAs) (6, 7). Pseudogenes are widely expressed in human cancers (8), and alterations in lncRNA gene expression levels are relevant for tumorigenesis (9). However, differences in the expression of lncRNA genes or pseudogenes between cervical SCC and ADC in the context of regulatory networks have not been described to date.

Thus, we aimed to unravel gene expression profiles that could discriminate different CC histological types in the context of transcriptional regulatory networks (TRNs) of protein-coding and non-coding RNA genes, such as lncRNAs. Here, we identified differences in gene expression profiles between cervical SCC and ADC, which were further analyzed in the context of TRNs, unveiling TFs that participate in epithelial maintenance and the maturity-onset diabetes of the young (MODY) pathways, respectively. The identification of these dysregulated networks contributes not only to the molecular characterization of CC of different histological types but may also support investigations for the identification of specific biomarkers and the development of treatments.

## Materials and Methods

### Cohort Study

CC tumor biopsies were obtained from 30 untreated women with histologically proven cervical SCC (*n* = 25) or ADC (*n* = 5) referred to the Gynecological Service of the Instituto do Cancer do Estado de São Paulo (ICESP, São Paulo, Brazil) (Supplementary Table 1).

All patients signed an informed consent form before biopsies were obtained. Histological analysis was carried out using hematoxylin and eosin-stained sections at ICESP.

### DNA and RNA Isolation, and Human Papillomavirus Genotyping

According to the manufacturer's instructions, DNA and RNA were isolated from each cervical tissue sample using the DNA/RNA All Prep kit (Qiagen, Germany).

Isolated DNA concentration and purity were evaluated using a NanoDrop 2000 spectrophotometer (Thermo Scientific, USA), and samples were diluted to a final concentration of 50 ng/μl. HPV detection and genotyping were performed using 100 ng of each DNA sample and the Inno-LiPA HPV Genotyping kit (FujiRebio, Belgium), according to manufacturer instructions.

RNA isolation was followed by DNase treatment. RNA integrity and quality were evaluated using the 2200 Tape Station System (Agilent Technologies Inc, USA), and RNA integrity number ≥ 8 was considered the quality threshold. In total, seven cervical SCCs and three ADCs with < 20% necrotic tissue were submitted to RNA sequencing (Supplementary Table 1).

### RNA Sequencing

All qualified CC samples were submitted to library preparation using the TruSeq Stranded messenger RNA (mRNA) LT Sample Prep kit (Illumina Inc., USA) and paired-end (2 × 100 bp) sequencing using the HiSeq SBS Kit v4 and the HiSeq Flow Cell v4 with HiSeq 2500 system (Illumina Inc., USA) at the Centro de Genômica Funcional Esalq—University of São Paulo. On average, 85 million paired-end reads were sequenced per tumor sample (Supplementary Table 2).

### Public Cervical Gene Expression Data Included in This Study

Transcriptome data of HPV-negative non-tumoral cervical samples (named non-CC) were downloaded from the Gene Expression Omnibus portal (accession PRJNA454568) (10).

Because gene-gene interaction analysis must be performed with a sample size bigger than the one used in this study, we used The Cancer Genome Atlas (TCGA) biolinks to extract data from the cervical tumors (TCGA-CESC). We downloaded 280 CC transcriptome data from the public TCGA repository under the tag TCGA-CESC, including 249 cervical SCC and 31 cervical ADC samples. We further compared gene expression profiles from our study samples with the TCGA dataset (Supplementary Table 3).

### Transcriptome Bioinformatic Analysis

RNA sequence data output (FASTQ files) was trimmed with Trim Galore (v. 0.0.4.0) (11) and aligned onto the GRC37/hg19 version of the human genome and the HPV-16 genome (NC_001526.4, GenBank) using Hisat2 (v. 2.1.0) (12) with the following parameters: “--dta --fr -q --no-mixed --no-discordant”. The latest human gene annotation dataset (hsapiens_gene_ensembl, available at http://grch37.ensembl.org) was obtained using the biomaRt package (v. 2.34.2) (13) from R statistical environment. SAM files were converted, merged, sorted, and indexed using Samtools utilities (14). The aligned reads were visualized using the IGV software (v. 2.3.82) (15). Reads counts were accessed using HTseq (v. 0.5.4p3) (16) applying the following parameters: “-m intersection-noempty -i id_gene -s reverse.”

Differential gene expression analysis was performed to compare between the normalized and filtered read count among three groups: cervical SCC, ADC, and non-CC (10) using the Bioconductor package edgeR (v. 3.20.9) (17), setting the log_2_ fold change module (|log_2_FC|) ≥ 2 and false discovery rate as ≤0.01. To overcome the batch effect due to combining data from independent cohorts, we used edgeR package and BatchEffect function from Limma Package, available at the Bioconductor project. Non-expressed and weakly expressed genes were defined as having ≤1 count (read) per million in *n* of samples in the smallest group for each comparison and were excluded from the differential expression analysis.

### Regulatory Networks Derived From Gene Expression Profiles

The regulatory network for both cervical SSC and ADC was inferred by computing mutual information between annotated TFs and all potential target genes using the RTN package (18). In the network architecture inferred, each TF was assigned to a list of candidate targets, which could be linked to multiple TFs. As regulation can occur from both direct (TF–target) and indirect interactions (TF–TF–target), the Algorithm for the Reconstruction of Accurate Cellular Networks was used as an additional step to enrich the regulatory networks with direct TF–target interactions (19). The resulting regulatory networks were plotted with Cytoscape (v. 3.7.2) (20).

### Gene Function Annotation and Pathway Enrichment Analysis

Enrichment of Gene Ontology biological processes of cervical SCC and ADC gene expression profiles were investigated using Enrichr (21). The GSEA software (version 4.0.3.4) (22) was used to identify Kyoto Encyclopedia of Genes and Genomes (KEGG) pathways showing an overrepresentation of up- or downregulated genes in cervical ADC or SCC. Briefly, an enrichment score was calculated for each gene set (i.e., KEGG pathway) by ranking each gene according to their expression difference using Kolmogorov–Smirnov statistic, computing a cumulative sum of each gene ranked in each gene set and recording the maximum deviation from zero as the enrichment score.

### Quantitative Real-Time Polymerase Chain Reaction Validation and Analysis

To validate genes with different expression levels revealed by whole RNA sequencing, we performed quantitative polymerase chain reaction (qPCR) of six selected differentially expressed lncRNA using RNA obtained from 20 cervical SCC and 5 cervical ADC samples (Supplementary Tables 1, 4). We selected for validation by qPCR lncRNAs with the highest or lowest log_2_FC values. Complementary DNA synthesis from 500 ng of total RNA isolated from CC samples was performed using the SuperScript VILO kit (Life Technologies, USA). qPCR was carried out using the SYBR Green PCR Master Mix on an ABI 7500 real-time PCR system (Applied Biosystems, USA). LncRNAs transcript levels were normalized to the mRNA levels of the ribosomal protein lateral stalk subunit P0 (*RPLP0*) constitutive gene. The lncRNA gene annotation from LNCipedia (23), Ensembl transcript identification, qPCR pair primers pair sequences, and expected amplicon length are available in Supplementary Table 4. The Mann–Whitney statistical test was applied to compare cervical SCC and ADC distribution, considering α = 0.01 as the significance level. Statistical analyses were performed using GraphPad Prism version 7 for Windows (24) and ggplot2 package (25) in the R statistical environment.

### *In silico* Analysis of MicroRNA–Messenger RNA Target Predictions

The following databases of miRNA targeting mRNA predictions have been investigated to detect potential mRNA transcripts regulated by the miR-28-3p or miR-96-5p: mirDIP with the minimum score set to medium (26); mirMap with the default parameters (27); TargetScan with a parameter to search for conserved sites for miRNA families conserved only among mammals (28); miRDB with the default parameters (29); and DIANA-microT with a threshold set to 0.5 (30). If miR-28-3p or miR-96-5p returned any positive result for the TF genes in the MODY pathway, a bidimensional matrix was populated (Supplementary Table 5).

### Development and Evaluation of a Cervical Adenocarcinoma Classifier

We developed a classifier based on TCGA data, considering the ADC histological type (possible responses: yes or no, when it was an SCC case) as our outcome of interest. According to differential expression analysis, the set of predictors was composed of those genes identified as enriched in cervical ADC gene profile followed by TRN analysis. We applied logistic regression to adjust the classifier and leave-one-out cross-validation (CV) technique to evaluate its performance in data not used for its adjustment. Briefly, on each CV iteration, the observations are randomly divided into a training and a test set, then we adjust a logistic model in the training data and estimate the risk (probability) of being classified as ADC on the test data. The predictive performance was evaluated by calculating the area under the receiver operating characteristic (AUROC) curve with 95% confidence intervals. Sensitivity (S), specificity (E), positive predictive value (PPV), and a negative predictive value (NPV) were measured for different estimated probability (p) cutoff points: one that maximizes model's sensitivity and specificity, and other aiming to improve model's specificity and, accordingly, to reduce the false-positive results (i.e., an SCC case classified as ADC). The latter approach was based on a cutoff point that distinguishes the 10% of the observations with the highest predicted risk for ADC. The adjusted model was also applied to our dataset, and S, E, PPV, and NPV were estimated according to the cutoff points described earlier. Analyses were performed using R software. Model training was carried out using the *caret* (Classification and Regression Training) package and model evaluation through pROC and ROCR packages.

## Results

Primary frozen tumor biopsies obtained from 30 treatment-naive women diagnosed with CC (25 SCCs and 5 ADCs) referred to the ICESP in São Paulo, Brazil, were submitted to HPV detection and typing (Supplementary Table 1). HPV was detected in 23 (92.3%) of the cervical SCC samples, and HPV-16 was the most frequent type found in 13 of 23 SCC samples. Co-infections by two HPV types were found in three SCC samples. In contrast, HPV-16 was the only type identified in all ADC cases (Supplementary Table 1). To investigate the gene expression profiles found in the two histological cancer types without the interference of different HPV types, the transcriptomes of seven SCC and three ADC HPV-16-positive samples were evaluated by RNA-Seq (Supplementary Table 1). As expected, viral E6 and E7 transcripts were detected in all samples submitted to RNA-Seq (Supplementary Table 2).

**Figure 1:**
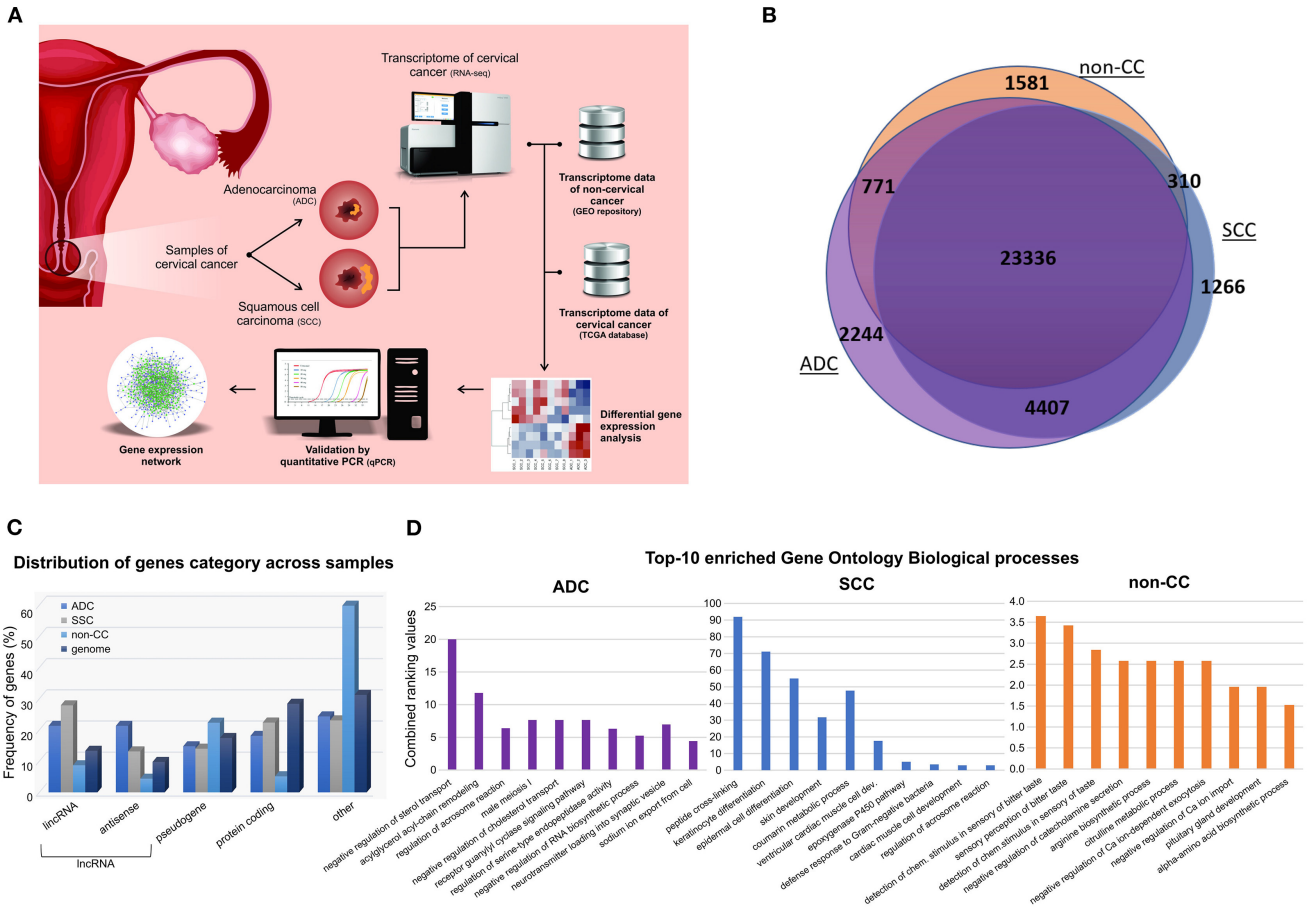
General design and descriptive results of cervical cancer transcriptome analysis. **(A)** Pipeline applied in cervical squamous cell carcinoma (SCC) and cervical adenocarcinoma (ADC) samples from our study, followed by inclusion of publicly available HPV-negative non-tumoral cervical tissue (non-CC) samples [10] and cervical SCC and ADC samples from The Cancer Genome Atlas (TCGA). Gene expression of SCC, ADC, and non-CC samples was evaluated. Expression levels of selected long non-coding RNA (lncRNA) genes differentially expressed between cervical ADC and SCC were validated by quantitative real-time PCR. Gene expression networks were predicted using gene expression profiles. **(B)** Number of genes expressed in SCC and ADC samples from this study and non-CC samples. **(C)** Frequency of genes expressed in cervical SCC, ADC, and non-CC samples, according to protein-coding, lncRNAs genes, and pseudogenes categories (x-axis). LncRNA genes were subdivided in antisense and long intergenic non-coding RNA (lincRNA), according to Ensembl database annotation (GRCh37/hg19 version). **(D)** Bar chart of top 10 enriched Gene Ontology biological processes based on gene expression profiles of cervical ADC and SCC samples from our study and non-CC samples, according to combined ranking values (y-axis) calculated by Enrichr (11).

The transcriptome data obtained from our samples were compared with four HPV-negative non-tumoral cervical tissues (hereafter nominated non-CC) downloaded from the Gene Expression Omnibus repository (PRJNA454568) (10). Figure 1A depicts the strategy outlined in our study. Overall, 33,915 expressed genes were identified, of which 25,998, 29,319, and 30,758 were in non-CC, SCC, and ADC samples, respectively. Although most genes (23,336/33,915; 68.81%) were found to be expressed within all sample groups, almost a quarter (7,917/33,915; 23.34%) were exclusively expressed in SCC or ADC or in both CC histological types (Figure 1B). In non-CC samples, 25,998 genes were expressed among the 33,915 (76.66%) genes observed among all groups. In addition, 86.45% (29,319/33,915) and 90.69% (30,758/33,915) of the genes were expressed among cervical SCC and ADC samples, respectively. Among all genes identified within each group, ADC samples harbored the highest proportion of genes exclusively expressed in these tumors (2,244/30,758; 7.30%), followed by non-CC (1,581/25,998; 6.08%), and SCC (1,266/29,319; 4.32%).

The non-CC, SCC, and ADC samples expressed distinct sets of protein-coding (mRNA), lncRNA genes, and pseudogenes in comparison with the frequency of these gene categories within the human genome (Figure 1C). Next, we sought to investigate the enrichment of Gene Ontology biological processes of expressed genes in each group (Figure 1D, Supplementary Figure 1). Pathways related to epithelial differentiation and lipid metabolism were identified in SCC and ADC, respectively. The distinct sets of genes reflect the different biological processes occurring in each histological tumor type.

Differential expression analysis was performed between SCC and ADC samples and SCC or ADC *vs*. non-CC samples. According to our analysis, we identified 135 upregulated and 258 downregulated genes in SCC as compared with ADC samples: 1,413 genes upregulated in SCC as compared with non-CC and 1,260 genes upregulated in ADC as compared with non-CC (Supplementary Tables 6–8, Supplementary Figures 2, 3).

**Figure 2:**
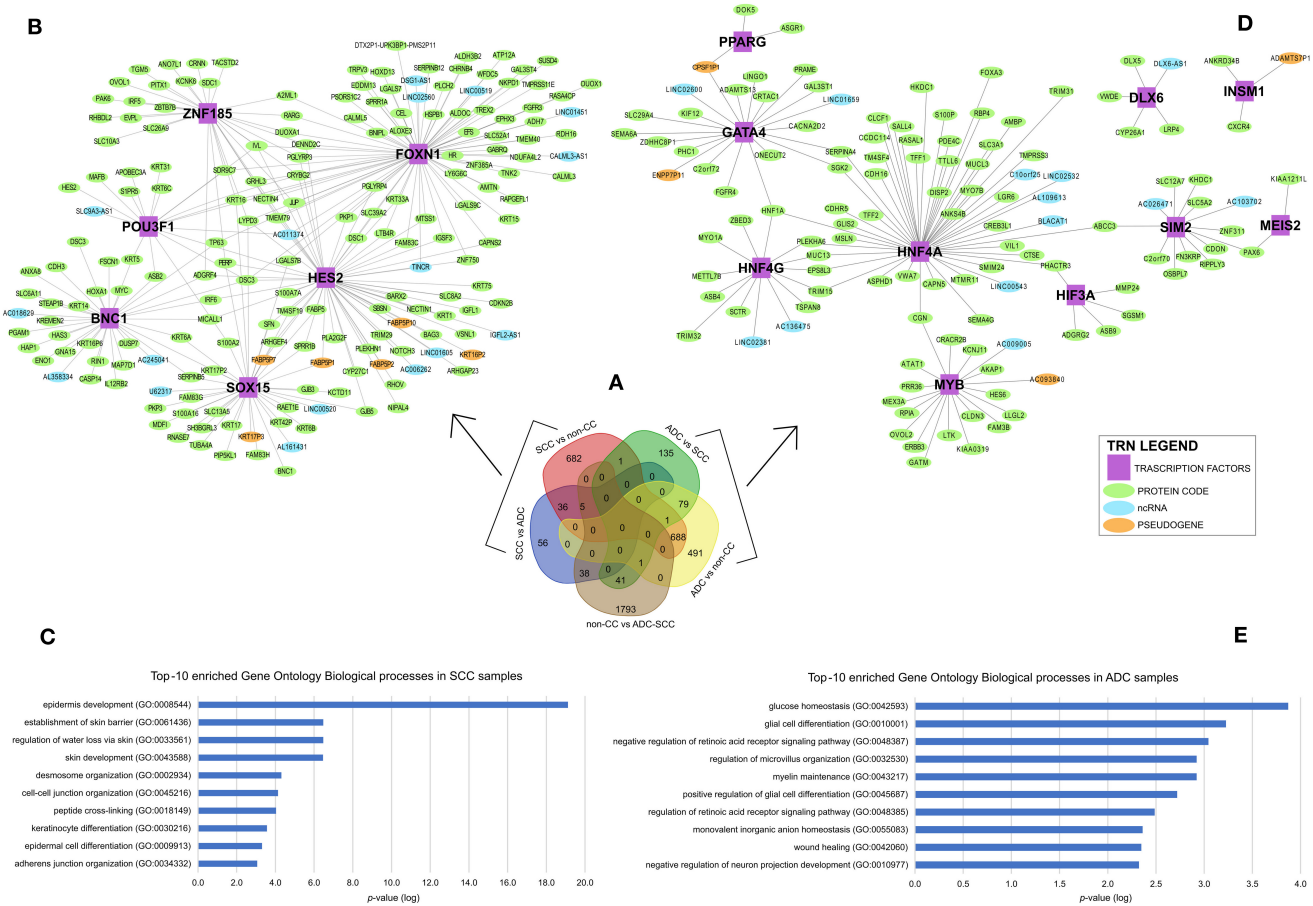
Differential gene expression profiles among cervical squamous cell carcinomas (SCC), cervical adenocarcinomas (ADC), and non-tumoral cervical tissue and HPV-negative (non-CC) samples. **(A)** Number of genes differentially expressed in each group. Transcription regulatory networks (TRN) of genes upregulated in SCC **(B)** and ADC **(D)**, including transcription factors (purple squared), other protein-coding genes (green circle), long non-coding RNAs (lncRNA) (light blue circle), and pseudogenes (light orange circle). Ten most enriched Gene Ontology Biological processes involved in cervical SCC **(C)** and ADC **(E)** TRNs. Biological processes were grouped by enriched Gene Ontology (GO) Biological processes (y-axis) and sorted by lower logarithm scale of *p-*values (x-axis, *p* < 0.05), calculated by Enrichr (11).

To identify gene expression patterns that might discriminate cervical SCC from ADC, we selected only those genes that were differentially expressed exclusively in each group or less expressed in non-CC samples, as shown in Figure 2A. The selected genes were investigated in the context of TRNs. This analysis identifies expression enrichment of specific TFs as key regulators of various target genes. However, due to our samples' small sample size and the need to use more than 100 samples to perform TRN analysis, we used only the TCGA data for this analysis. It is noteworthy that the differential gene expression profiles observed between cervical SCC and ADC in our study correlated to the respective profiles derived from the TCGA dataset with a Spearman's rank correlation coefficient of 0.79 (Supplementary Figures 3, 4, Supplementary Table 3).

Overall, 774 genes exclusively upregulated in SCC samples (682 genes from SCC *vs*. non-CC, 56 genes from SCC *vs*. ADC, and 36 overlapped genes from both SCC *vs*. ADC and SCC *vs*. non-CC) and 705 genes exclusively upregulated in ADC samples (135 genes from ADC *vs*. SCC, 491 genes from ADC *vs*. non-CC, and 79 overlapped genes from both ADC *vs*. SCC and ADC *vs*. non-CC) were used for the construction of TRNs (Figure 2A).

The distinct TRNs for SCC (Figure 2B) and ADC (Figure 2D) reflect biological processes enriched in each CC histological type, according to the Gene Ontology annotation (Figures 2C,E). The cervical SCC TRN showed *FOXN1, POU3F1, SOX15, ZNF185, HES2*, and *BNC1* TF genes with the potential to positively regulate several other protein-coding genes, lncRNAs, and pseudogenes (Figure 2B) that are related to cornification, keratinization, and epithelial differentiation processes (Figure 2C). Ten TFs within the cervical ADC TRN analysis, *PPARG, GATA4, HNF4G, DLX6, INSM1, MYB, HIF3A, SIM2, MEIS2*, and *HNF4A* (Figure 2D), are related to carbohydrate and glucose homeostasis processes (Figure 2E).

**Figure 3:**
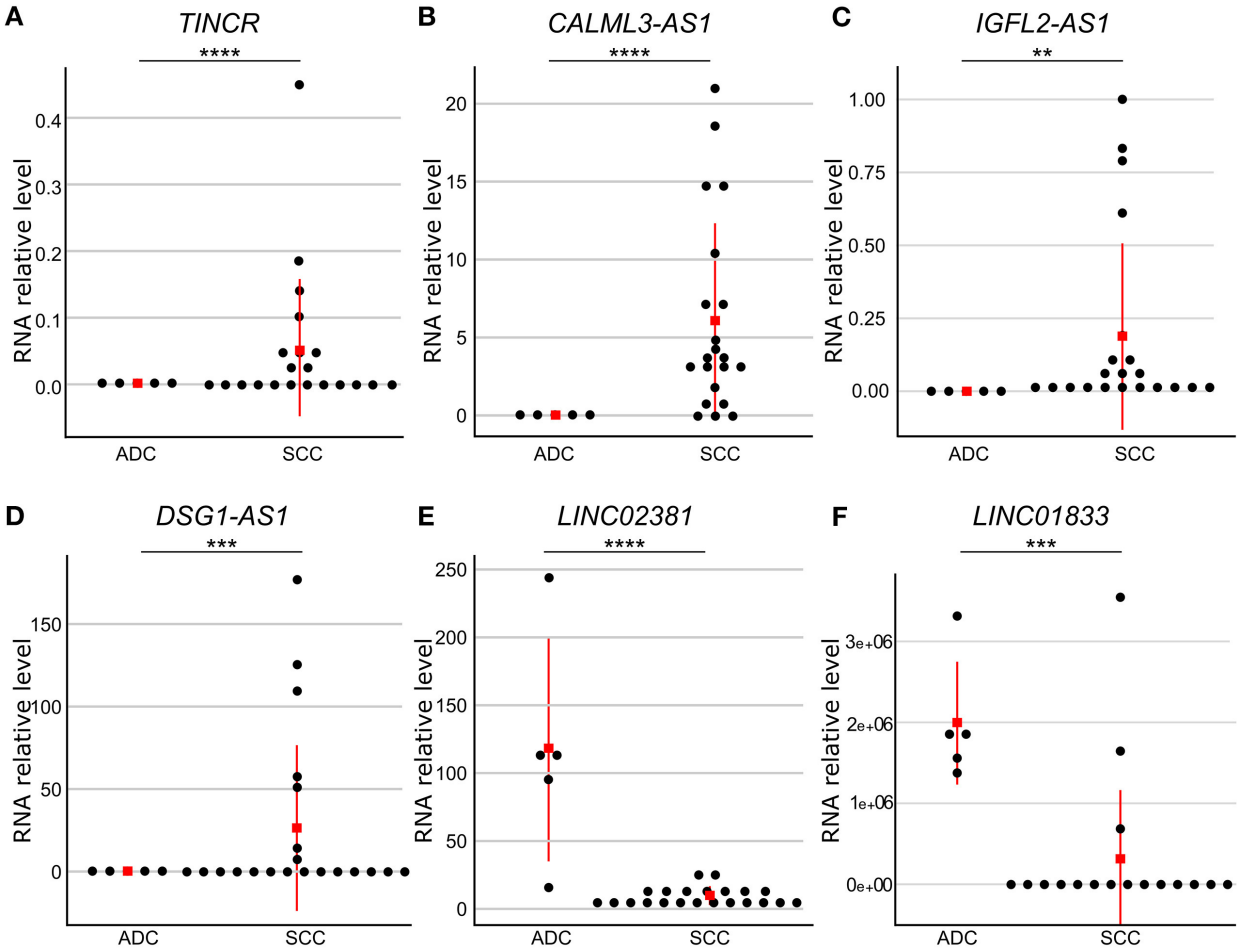
Gene expression levels of six differently expressed long non-coding RNA (lncRNA) genes between cervical adenocarcinoma (ADC) and squamous cell carcinoma (SCC). RNA levels were evaluated by quantitative reverse transcriptase-polymerase chain reaction (qPCR) and normalized to RPLP0 gene levels in SCC (*n* = 25) and ADC (*n* = 5) samples. Red square indicates median value. Gene expression levels were significantly different using Mann–Whitney test (*p* ≤ 0.05). *TINCR* **(A)**, *CALML3-AS1* **(B)**, *IGFL2-AS1* **(C)**, and *DSG1-AS1* **(D)** lncRNA genes presented higher expression levels in cervical SCC as compared with ADC, whereas *LINC02381* **(E)** and *LINC01833* **(F)** lncRNA genes were upregulated in ADC as compared with SCC samples. *p-*values are indicated as **p < 10^−2^, ***p < 10^−3^, ****p < 10^−4^.

To confirm the observed differences in gene expression levels obtained from the differential expression analysis of our RNA-seq data, we selected six lncRNA genes differently expressed between cervical SCC and ADC for validation in a larger number of samples (Supplementary Tables 4, 6). qPCR results revealed that *DSG1-AS1, CALML3-AS1, IGFL2-AS1*, and *TINCR* genes were upregulated in cervical SCC as compared with ADC. Interestingly, these lncRNA genes are regulated by FOXN1 and HES2 TFs. *LINC02381* and *LINC01833* lncRNA genes were upregulated in cervical ADC as compared with SCC, corroborating our transcriptome results (Figure 3, Supplementary Table 6).

**Figure 4:**
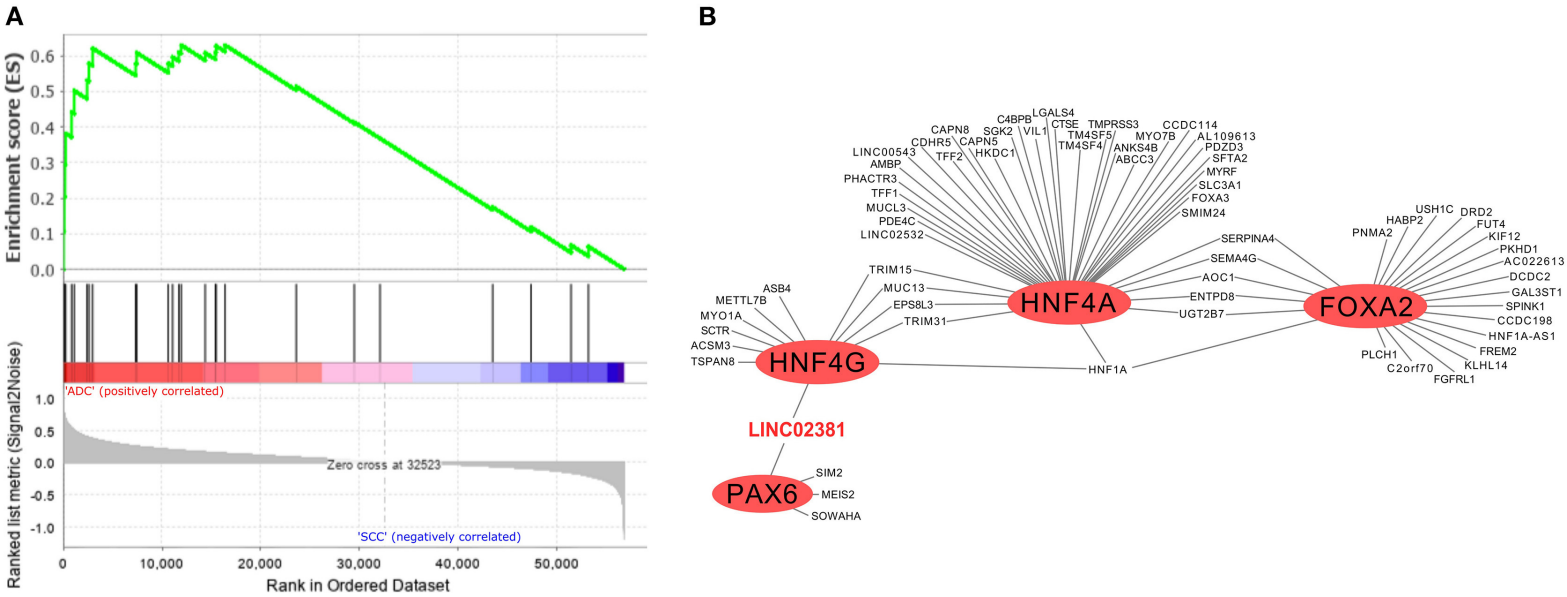
Gene set enrichment analysis and transcriptional regulatory network (TRN) of upregulated genes in cervical adenocarcinoma (ADC) samples from our study. **(A)** Fourteen genes enriched in ADC that participate in maturity-onset diabetes of the young (MODY) pathway were not expressed neither in SCC nor in non-tumoral cervical tissue and HPV-negative (non-CC) tissue samples, calculated by GSEA software (12). **(B)** Among transcription factors (TF) related to MODY pathway, hepatocyte nuclear factor 4 (*HNF4G/A*), pancreatic and duodenal homeobox 1 (*PDX1*), and forkhead box protein A2 (*FOXA2*) (red circles) are related to expression of several upregulated genes in ADC samples in TRN analysis, including protein-coding genes and of *LINC02381* long non-coding RNA (lncRNA) gene (in red).

In addition to the biological processes observed in SSC and ADC samples (Figure 2), we investigated enriched biological pathways using the KEGG database based on genes exclusively expressed in each CC histological type (Supplementary Table 9). Several pathways were enriched in SCC, such as the estrogen signaling pathway (KEGG ID map04915; Supplementary Figure 5), whereas the MODY pathway was enriched in ADC (Figure 4A, Supplementary Figure 6). At last, we filtered all TFs from the MODY pathway (Supplementary Table 10) together with upregulated genes detected in the TRN of ADC to obtain a MODY-specific TRN in ADC. This analysis identified *HNF4A, HNF4G, FOXA2*, and *PAX6* TF genes in cervical ADC TRN, including a set of protein-coding and lncRNA genes, such as the *LINC02381* gene (highlighted in red in Figure 4B).

**Figure 5:**
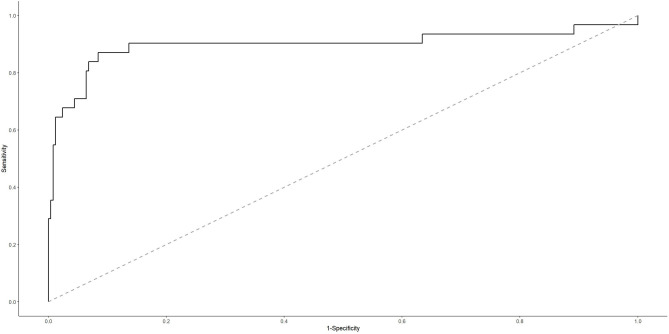
Area under the receiver operating characteristic (AUROC) curve for cervical ADC classifier.

To evaluate the potential of genes encoding MODY pathway TFs in discriminating between ADC and SCC CC types, we adjusted a classifier considering the ADC histological type (possible responses: yes or no, when it was an SCC case) as our outcome of interest and the set of genes *HNF4A, HNF4G, FOXA3, PAX6, HNF1A, PDX1*, as well as the lncRNA *LINC02381*, as our predictors. For each iteration of the leave-one-out CV process, the adjusted classifier was evaluated in the test data. At the end of the CV process, the estimated risk from iterative test data was aggregated to estimate the AUROC curve, which results indicate a good discrimination performance for our classifier (AUROC curve: 0.8978; 95%; confidence interval: 0.808–0.987) (Figure 5).

Two scenarios were defined according to the cutoff points we adopted for the estimated probability. For the cutoff point that maximizes model's sensitivity and specificity (*p* ≥ 0.077), it is possible to successfully predict 87.10% (27 of 31 cases) of ADC cases. Nonetheless, it is important to note that, although the negative predictive value is close to 1 (that is, when the classifier indicates an SCC case, it is almost always correct) because ADC is a rare event, the positive predictive value is low (if the classifier indicates an ADC case, then further investigation is necessary because, in half of the times, it is a false result). So, this would be a good classifier to rule out the possibility of SCC, requiring as a next step some approach to confirm ADC. Additionally, aiming to improve the model's specificity and, accordingly, to reduce false-positive results, we chose a cutoff point that might discriminate those patients in the 10% highest risk strata (*p* ≥ 0.2082). In this scenario, it is possible to successfully predict 67.74% (21 of 31 cases) of ADC cases. Additionally, because the false-positive rate has reduced from 8 to 3% (in comparison with the first scenario we described), now the positive predictive value is 75.00%. This classifier would be a good tool when we want to be more confident about the classifier result because, for example, it is necessary to prioritize a group (higher risk ADC group) for some intervention (Supplementary Table 11).

Considering the former or the latter cutoff point for classifying our study data led to the same results: S, E, PPV, and NPV were, respectively, 0.667, 1.00, 1.00, and 0.875. Of the three ADC cases, two presented an estimated probability higher than that of the SCC cases; however, one of the ADC samples had an estimated probability of 0.016 (below the two cutoff points described earlier), and it was labeled as SCC (a false-negative result).

## Discussion

Differences in cervical SCC and ADC transcriptomes have been previously investigated, focusing on protein-coding genes. Additionally, functional network analysis (31) and drug repurposing for cervical ADC or cervical SCC have been proposed (32, 33). However, no set of TFs and lncRNAs coordinately regulated with the potential to explain cervical ADC carcinogenesis have been described to date. In this study, we confirmed the upregulation of protein-coding genes previously identified in ADC, such as *IGFBP2* (34), and in SCC, we identified *CDKN2A*, which encodes p16 (35), and *E2F1* (36).

Furthermore, we describe TRNs that discriminate between HPV-16-positive cervical SCC and ADC and between both histological types and non-CC tissues. Genes found upregulated among SCC samples are involved in biological processes related to epithelium structure and function, including maintenance of skin barrier due to epithelium attachment, in addition to cornification and keratinization processes. We observed the enrichment of keratin coding genes highly expressed in cervical SCC, similar to previous data of the TCGA consortium (34). On the other hand, the highly expressed genes among cervical ADC samples are associated with carbohydrate and glucose homeostasis.

It is important to note, however, that one limitation of this study is that we used previously published RNA-Seq data from normal cervical tissues (non-CC), which may have a distinct genetic background compared with the CC samples sequenced here. Furthermore, the restricted sample number unable us to discriminate whether the genes are differentially expressed between tumor and non-tumor samples results from HPV-16 infection. Nevertheless, despite the few cervical ADC samples included in this study, we believe that our data enabled the identification of biological signals to computationally discriminate cervical SCC from ADC. Such promising findings must be further confirmed and investigated in depth by additional studies.

### Epithelium Structure and Function Pathways Are Enriched in Cervical Squamous Cell Carcinoma Gene Profile

The upregulation of genes involved in epithelium maintenance among cervical SCC samples was further endorsed by the TRN analysis, which identified regulons whose lncRNA genes are highly expressed in SCC in contrast to ADC or non-CC samples. Epithelial differentiation and homeostasis are governed by several TFs (37–39). Among our findings, POU Class 3 Homeobox 1 (*POU3F1*) (40), Basonuclin 1 (*BNC1*) (41), Human Forkhead-box N1 gene (*FOXN1*) (42), hes family bHLH transcription factor 2 (*HES2*) (43), and zinc finger protein 185 with LIM domain (*ZNF185*) (44) were previously described to be involved in keratinocyte differentiation, or skin development, homeostasis, and wound healing.

One important regulator of the expression of several genes involved in keratinocyte proliferation and differentiation is p63 (45), which is encoded by the tumor protein p63 (*TP63*) gene. We found *TP63* upregulated in cervical SCC as compared with ADC and non-CC (Supplementary Table 6, Figure 2B). Indeed, p63 was previously detected by immunohistochemistry in 97% of cervical SCC, whereas it was absent in ADC (46). In this study, *TP63* integrates the SCC TRN as a gene potentially regulated by *BNC1* and *FOXN1*. Expression of *BNC1* and *ZNF185* have been previously described to be positively regulated by p63 (47, 48). Higher expression levels of *BNC1* and SRY-Box Transcription Factor 15 (*SOX15*) genes have also been previously detected in SCC samples from other anatomical sites compared with their related non-tumoral tissues (49–51). In contrast, *ZNF185* displayed a discordant expression pattern in cervical SCC compared with our findings (48). However, because the expression of the protein encoded by this gene was shown to vary widely among skin layers (44), we suggest that our findings must be investigated in-depth in the future. Two of the six TFs within the cervical SCC TRN were also previously reported to be positively regulated by p63. We thus conclude that the cervical SCC TRN proposed in the present study encompasses a set of TFs with functions associated with epithelial differentiation and homeostasis, supporting its role in cervical SCC.

In addition to the putative role of different TFs upon cervical SCC TRN, several lncRNAs genes regulated by these proteins might also affect integrity maintenance and differentiation of the epithelium. Using qPCR, we validated the higher expression of TINCR ubiquitin domain-containing (*TINCR*), CALML3 antisense RNA 1 (*CALML3-AS1*), DSG1 antisense RNA 1 gene (*DSG1-AS1*), and IGF-like family member 2 antisense RNA 1 (*IGFL2-AS1*) lncRNA genes in cervical SCC in comparison with cervical ADC samples. Nevertheless, it needs to be highlighted that, to date, the functions of most of the cataloged human lncRNA are not clarified due to the lack of specific literature.

Increased *TINCR* lncRNA levels have been found within differentiated superficial epidermis layers and were shown to positively regulate the expression of the MAF:MAFB TF dimer (52). Corroborating our findings, higher expression of *TINCR* in cervical SCC was previously observed (53), although these data contrast with data obtained from TCGA (54). All other validated lncRNA genes in our study were classified as antisense transcripts (55). We observed higher expression levels of the *IGFL2-AS1* in cervical SCC compared with cervical ADC and non-CC. Higher levels of this antisense gene have been described in renal cell carcinoma (56) and gastric cancer (57); in contrast, a lower expression of *IGFL2-AS1* was observed in breast ADCs (58). The *CALML3-AS1* lncRNA was shown to increase proliferation, migration, and invasion of CC cells and to decrease the apoptosis of these cells. Indeed, high *CALML3-AS1* levels were previously observed in CC samples of TCGA (59). In our study, lower *CALML3-AS1* levels were detected in cervical ADC and non-CC compared with cervical SCC, indicating this molecule may be further explored as a molecular target for specific diagnosis of this histological tumor type.

In conclusion, although the function of most of the lncRNAs remains unclear, a selected set of such transcripts were successfully validated as highly expressed in cervical SCC as compared with ADC. Among them are *TINCR* and *CALML3-AS1*, whose role in CC is under investigation. Taken together, our data reinforce the molecular differences between cervical SCC and ADC, whose significance should be explored in the prognostic and therapeutic management of these tumors.

### Genes Encoding Maturity-Onset Diabetes of the Young Pathway Transcription Factors Are Enriched in Cervical Adenocarcinoma Gene Profile

ADC arises from the cervical glandular tissue and leads to abnormal epithelium structure and production of secreted molecules. In this study, we describe a set of 10 TF genes upregulated in ADC as compared with SCC or non-CC. All TFs within the ADC TRN have been previously associated with the genesis of ADCs, in particular pancreatic ductal adenocarcinoma (PDA), with the exception of the insulinoma-associated protein 1 (*INSM1*). Although no literature regarding *INSM1* in PDA, it was described to have a key role in pancreatic development (60), similar to the GATA binding protein 4 (*GATA4*) (61).

*GATA4* gene expression levels were shown to vary in ADCs arising from distinct tissues (62, 63). In PDA, GATA4 protein levels were detected with higher intensity in women than in men (64). In addition to *GATA4* gene expression, the peroxisome proliferator-activated receptor gamma gene (*PPARG*) (65), the hepatocyte nuclear factor-4-alpha (*HNF4A*) (66), the hypoxia-inducible factor 3 subunit alpha (*HIF3A*) (67), the MYB proto-oncogene (*MYB*) (68), and the single-minded 2 (*SIM2*) (69) genes were also previously described to be highly expressed in PDA. To our knowledge, this is the first report to identify the surprising similarity of TFs overexpressed in cervical ADC and PDA. Such similarity encourages additional investigations. Interestingly, although highly expressed in PDA, high levels of *SIM2* in cervical SCC have been associated with better overall survival compared with tumors expressing low levels of this gene (70). The Meis homeobox 2 (*MEIS2*) gene encodes for a TF that was identified as a key signal transduction pathway regulator in breast ADC (71). Recently, *MEIS2* was identified as highly expressed in cervical SCC and showed prognostic value (54). Therefore, we believe that additional studies must be carried out to clarify the possible roles of *SIM2* and *MEIS2* in the distinct histological types of CC.

The pathway enrichment analysis in cervical ADC unveiled the MODY pathway. MODY TFs have also been extensively investigated in the context of pancreas and liver development. In addition to *HNF4A* expression in PDA, *HNF4A* and *HNF4G* genes were reported to be positively regulated by HNF1 homeobox A (HNF1A) protein in the pancreatic cell (72). Corroborating our findings, *HNF1A* was shown to have higher expression levels in CC compared with the normal cervix (73), despite the fact that the histological type was not indicated in their study. Nevertheless, we may speculate that *HNF1A* was identified as differentially expressed in their study due to an overrepresentation of cervical ADC among samples. Another MODY pathway member, the gene encoding the TF pancreatic and duodenal homeobox 1 (*PDX1*), had the literature reviewed in the context of pancreas embryogenesis (74), including PAD (75). In an extensive analysis regarding pancreatic cancer, *PDX1, MNX1, HNF4G, HNF4A, HNF1B, HNF1A, FOXA2, FOXA3*, and *HES1* were proposed as the key TFs associated with the pancreatic progenitor subtype (76). Except for *FOXA2* and *HES1*, all of these TFs were identified to be overexpressed in cervical ADC (log_2_FC ≥ 2 and false discovery rate < 0.05; Supplementary Table 8), providing stronger support to the hypothesis that cervical ADC and PDA share molecular similarities. It is important to stress, however, that such relationships regarding cervical ADC regulation are predictions and must be further investigated and validated.

The construction of a TRN including all MODY TFs highly expressed in cervical ADC revealed *PAX6, HNF4A, HNF4G*, and *FOXA2* genes with the potential to positively regulate other protein-coding or lncRNA genes (Figure 4B). At the moment, the role of these TFs within the context of cervical ADC is unknown. However, the MODY pathway was previously associated with other ADCs, such as EAC and PAD. Our TRN analysis showed that *PAX6* and *HNF4G* TF genes might positively regulate the expression of the lncRNA *LINC02381* gene (also known as *LOC400043*). Although there is limited literature regarding *LINC02381*'s biological role, it was described to have a suppressive effect upon the tumorigenesis of human colorectal ADC (77). These data indicate that MODY TF genes may be associated with other molecular processes in addition to those previously described in onset diabetes. Additionally, we were able to validate that *LINC02381* and *LINC01833* had higher expression levels in cervical ADC than in cervical SCC by qPCR.

Although the role of lncRNAs in CC development is not completely elucidated, it is well-established that they can compete for binding to mRNAs with other regulatory molecules, such as miRNAs (78, 79). In the context of HPV-associated tumors, several studies indicate that E6/E7 viral oncoproteins target lncRNAs, subverting cellular processes seminal to carcinogenesis (80). Previous *in silico* miRNA to mRNA targeting predictions analyses, followed by experimental validation, identified that miR-96-5p and miR-28-3p could directly bind to LINC02381 (79).

**Figure 6:**
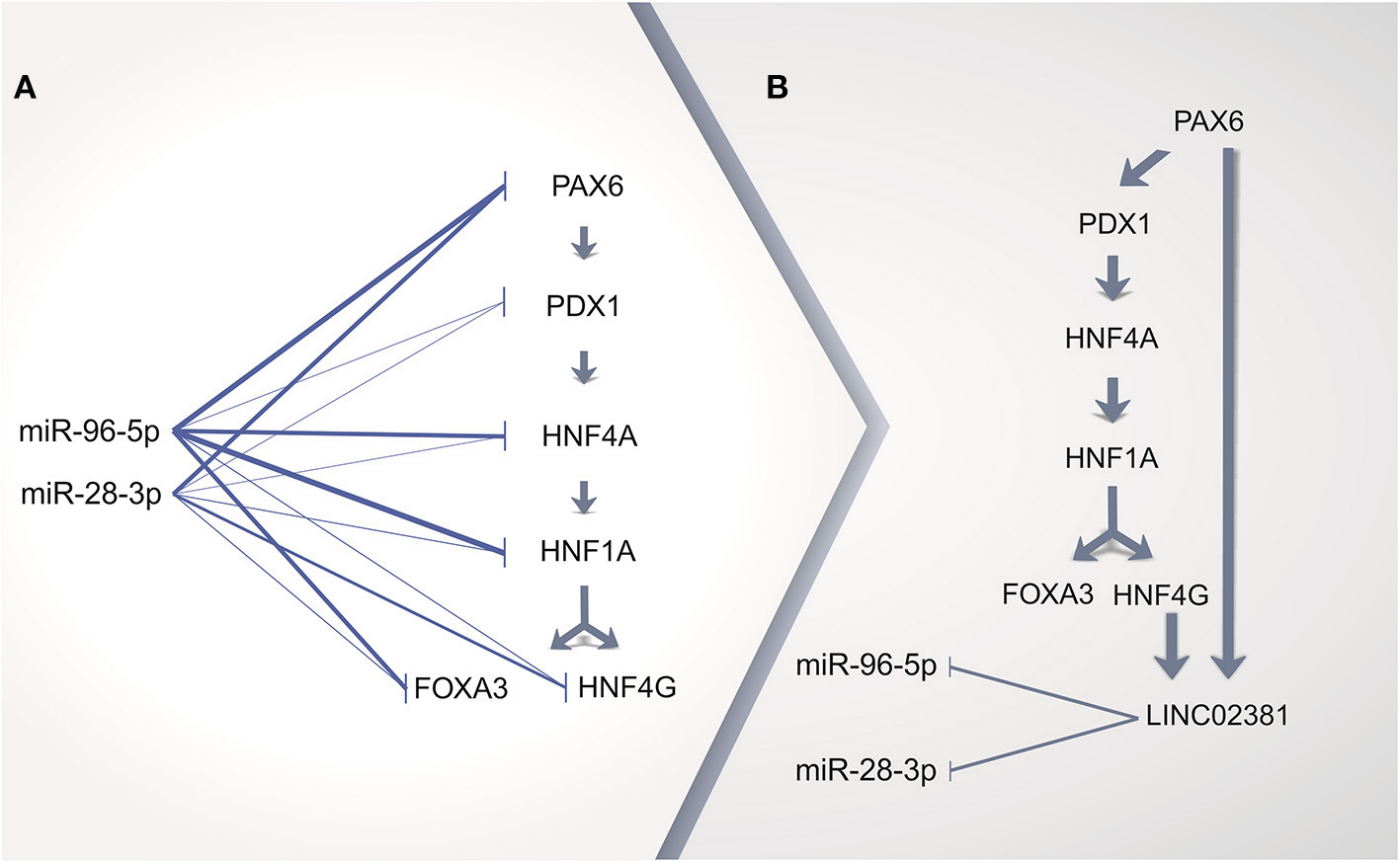
Proposed scheme of cervical adenocarcinoma (ADC) gene regulation by long non-coding RNA (lncRNA) acting as a sponge of microRNA (miRNA). **(A)** *in silico* prediction analysis of miRNA repressing all transcription factor (TF) genes upregulated in cervical ADC as compared with squamous cell carcinoma (SCC) in maturity-onset diabetes of the young (MODY) KEGG pathway. **(B)** TF genes HNF4G and PAX6 might upregulate LINC02381 lncRNA, which could act as a miRNA sponge, as positive feedback for all TF genes pathway expression. Thicker lines on **(A)** represent more positive predictions of miRNA targeting to a given mRNA (Supplementary Table 5).

The inspection of the entire MODY pathway (KEGG ID hsa04950) regarding the highly expressed TFs in cervical ADC allowed for the identification of the following set of MODY TF genes regulated in sequence: *PAX6, PDX1, HNF4A, HNF1A, HNF4G*, and *FOXA3* (Supplementary Table 10, Supplementary Figure 6). Surprisingly, according to at least one of five prediction tools, this set of TF genes within the MODY pathway is a potential target of either miRNAs miR-96-5p or miR-28-3p (Figure 6A, Supplementary Table 5). Interestingly, miR-96-5p has been associated with CC tumorigenesis progression by regulating the PTEN pathway (81).

Integration of MODY TRN and MODY TFs regulated in sequence in cervical ADC revealed that *HNF4G* and *PAX6* might lead to increased expression of *LINC02381*. We speculate that the enhanced expression of *LINC02381* in cervical ADC could dysregulate the repression role of miR-96-5p and miR-28-3p, acting as a sponge of such miRNAs (79). Therefore, the repressed MODY pathway previously silenced in the cervix would become active in cervical ADC (Figure 6B).

Taken together, our TRN analysis revealed a set of MODY TFs upregulated in cervical ADC. Among these, two TFs could positively regulate the lncRNA *LINC02381*. According to the literature, *LINC02381* can directly bind to two miRNAs acting as a sponge, reducing the availability of miR-96-5p and miR-28-3p. Computational predictions showed that miR-96-5p and miR-28-3p might negatively regulate this set of TFs. Therefore, we speculate *LINC02381* may act by decreasing the levels of miR-96-5p and miR-28-3p and promoting the upregulation of the MODY pathway in cervical ADC. Such findings, when considered as predictors in a classifier to differentiate ADC and SCC cases, resulted in encouraging results. Nonetheless, it is important to note that external validation, based on a larger dataset, is required to corroborate our results because, due to the small sample size (especially for the positive ADC type), we chose to use leave-one-out CV process and, therefore, effectively all data were used to estimate the predictive performance of the classifier, as this process implies a rotation of roles (training and testing) for each sample at each iteration performed.

## Conclusion

Cervical SCC and ADC patients frequently receive identical treatments. Therefore, it is urgent to identify novel therapeutic targets to treat cervical ADC. In the present study, we report distinct TRNs for cervical SCC and ADC. Further analysis uncovered the TF encoding genes *PAX6, PDX1, HNF4A, HNF1A, HNF4G*, and *FOXA3* with higher expression levels in cervical ADC as compared with cervical SCC or HPV-negative non-tumoral cervical tissues. This set of six TF genes is regulated in sequence in the MODY pathway. Based on bioinformatics predictions, we propose a hypothetical activation molecular mechanism of this set of six TFs, whose importance has been reported previously in pancreatic cancer. Our findings might provide novel directions to the development of more specific diagnostic tools for cervical ADC.

## Data Availability Statement

The datasets presented in this study can be found in online repositories. The names of the repository/repositories and accession number(s) can be found at: https://www.ncbi.nlm.nih.gov/, PRJNA521318.

## Ethics Statement

The studies involving human participants were reviewed and approved by Comitê de Ética em Pesquisa da Faculdade de Medicina da Universidade de São Paulo Av. Dr. Arnaldo, 251 - 21° andar - sala 36 Cerqueira César - São Paulo - SP - Brazil, CEP: 01246-000, +55 11 3893 4401, cep.fm@usp.br. This study was performed according to the Brazilian federal laws and approved by the Ethics Committee of the Faculdade de Medicina, Universidade de São Paulo (National Committee in Research Ethics, CONEP, number 53156815.5.0000.0065). The patients/participants provided their written informed consent to participate in this study.

## Author Contributions

PSA-S, LLV, LS, and FP: conceptualization. SB, TDJF, PSA-S, NANJ, GW, HGS, RC, LS, and FP: data curation. SB, TDJF, PSA-S, NANJ, HGS, RC, LS, and FP: formal analysis. LLV: funding acquisition. SB, TDJF, PSA-S, NANJ, HGS, MAAC, RC, LLV, LS, and FP: investigation. SB, TDJF, PSA-S, NANJ, HGS, MAAC, NMS, GW, RC, LLV, LS, and FP: methodology. MLNDG, JPC, LLV, and LS: sample acquisition. PSA-S, LLV, LS, and FP: supervision. SB, TDJF, PSA-S, and FP: writing—original draft. SB, TDJF, PSA-S, NANJ, LLV, LS, and FP: writing—review and editing. All the authors contributed to the manuscript revision, read and approved the submitted version.

## Conflict of Interest

LLV is speaker and consultant of Merck Sharp & Dohme for HPV prophylactic vaccines. The remaining authors declare that the research was conducted in the absence of any commercial or financial relationships that could be construed as a potential conflict of interest.
