# Supplementary material for: Dysregulation of Transcription Factor Networks Unveils Different Pathways in Human Papillomavirus 16-Positive Squamous Cell Carcinoma and Adenocarcinoma of the Uterine Cervix
Source: Front Oncol. 2021 May 19;11:626187. doi: 10.3389/fonc.2021.626187 (PMC8170088; doi:10.3389/fonc.2021.626187)
Supplement: Supplementary file 1 [file Image_1.pdf]

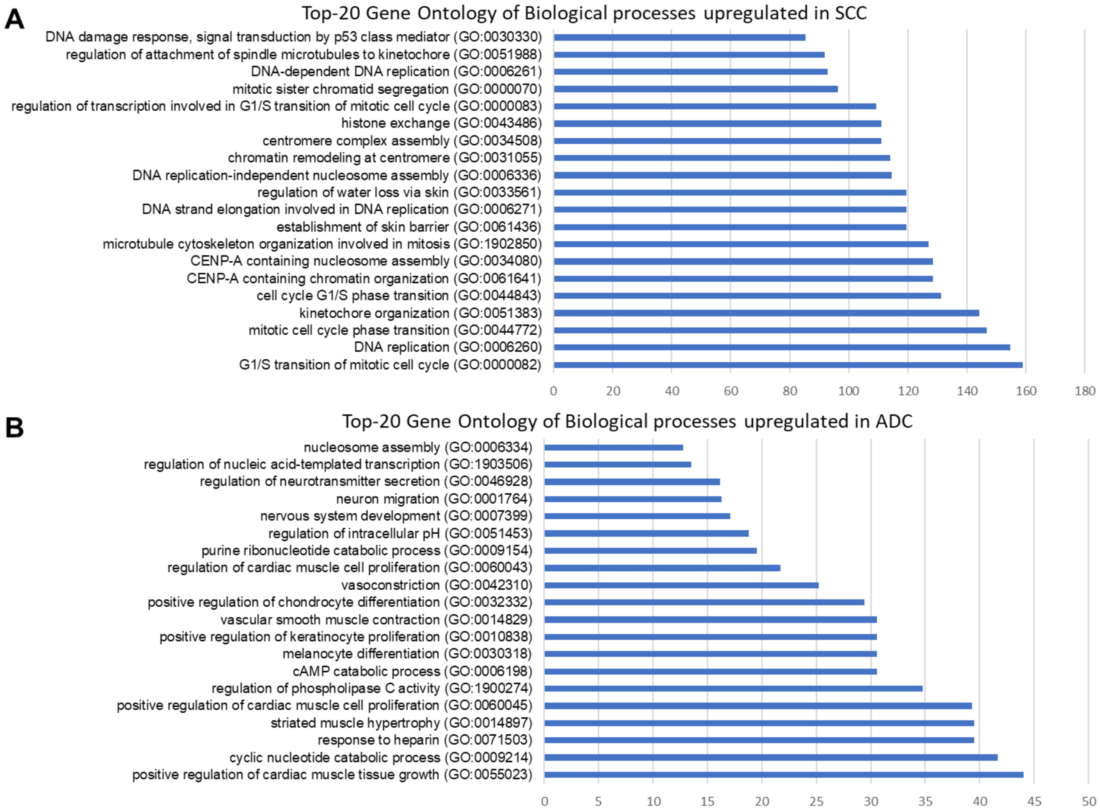

Supplementary Figure 1. Biological processes of upregulated genes in cervical SCC (A) or upregulated in ADC (B), based in Gene Ontology (biological processes) performed by Enrichr [11]. The top-20 combined ranking values of Biological processes (axis-x), p-value < 0.01.
