# Supplementary material for: Dysregulation of Transcription Factor Networks Unveils Different Pathways in Human Papillomavirus 16-Positive Squamous Cell Carcinoma and Adenocarcinoma of the Uterine Cervix
Source: Front Oncol. 2021 May 19;11:626187. doi: 10.3389/fonc.2021.626187 (PMC8170088; doi:10.3389/fonc.2021.626187)
Supplement: Supplementary file 2 [file Image_2.pdf]

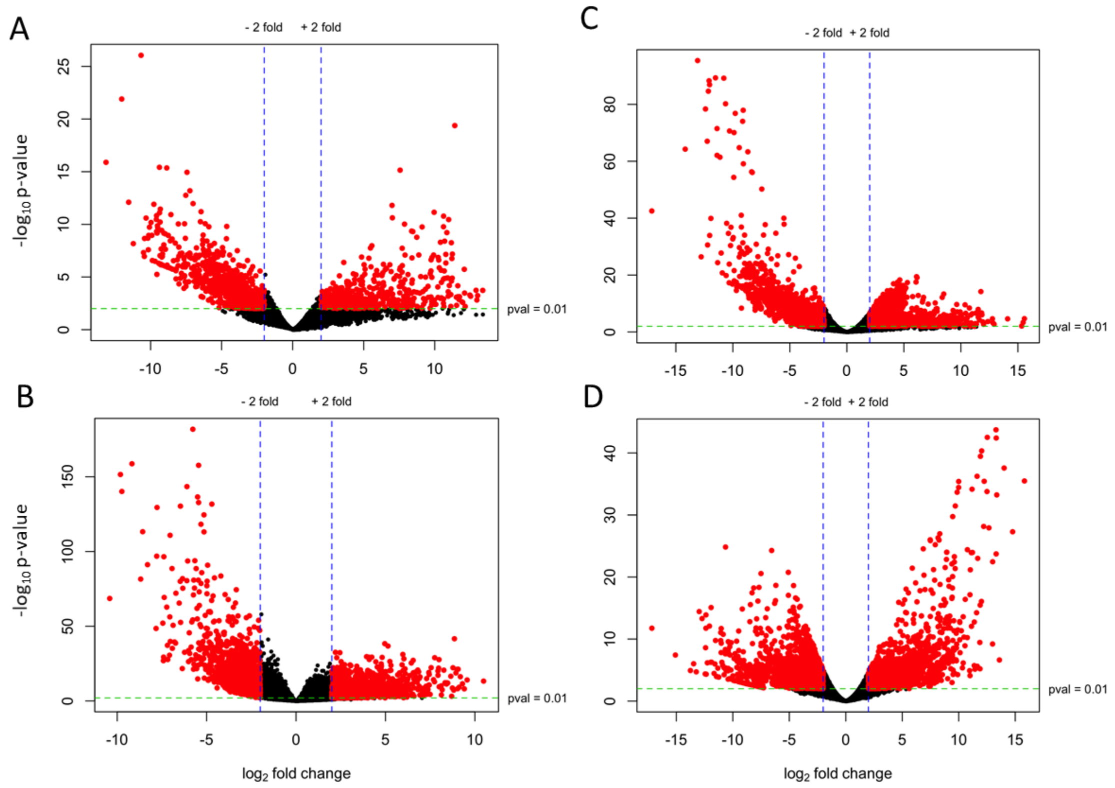

Supplementary Figure 2. Volcano plot of differentially expressed genes among different comparations of cervical samples: cervical squamous cell carcinoma (SCC), adenocarcinoma (ADC) from our study and from the TCGA, and non-cervical carcinoma cervix tissue (non-CC) [10]. Each dot is an expressed gene. In red, the differentially expressed genes with $|\log_2 FC| > 2$ (blue dashed line) and $p < 0.01$ (green dashed line). Differentially expressed genes between SCC and ADC in our samples (A), in TCGA samples (B), and SSC (C) and ADC (D) versus non-CC cervical samples.
