# Supplementary material for: Dysregulation of Transcription Factor Networks Unveils Different Pathways in Human Papillomavirus 16-Positive Squamous Cell Carcinoma and Adenocarcinoma of the Uterine Cervix
Source: Front Oncol. 2021 May 19;11:626187. doi: 10.3389/fonc.2021.626187 (PMC8170088; doi:10.3389/fonc.2021.626187)
Supplement: Supplementary file 3 [file Image_3.pdf]

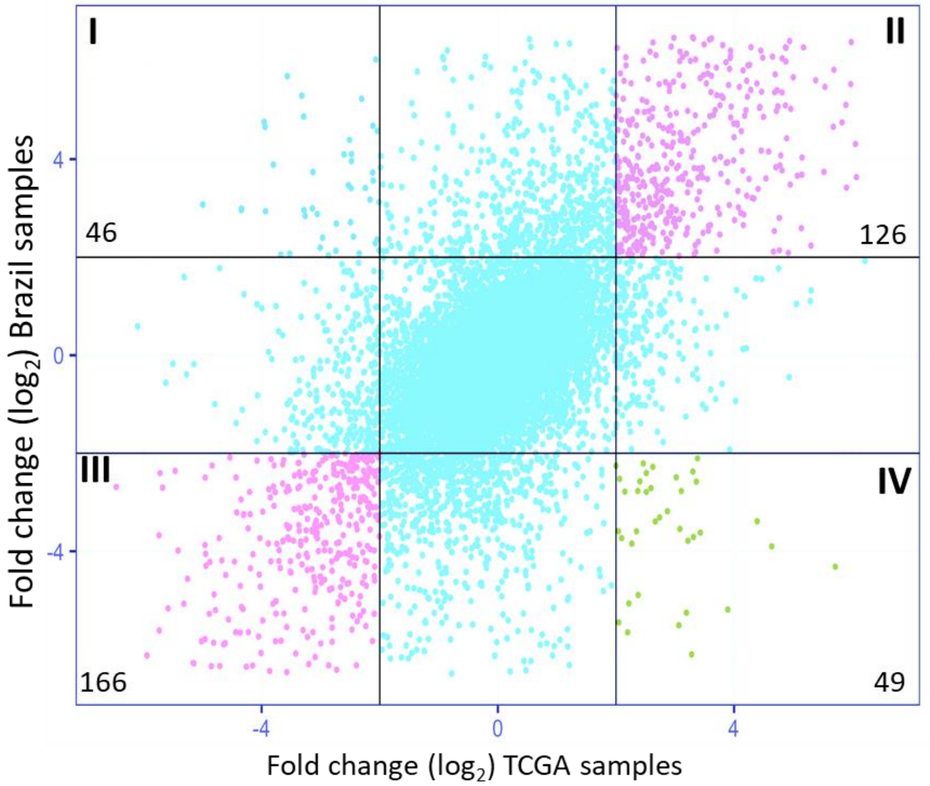

Supplementary Figure 3. Scatter plot shows the expression signature of differentially expressed genes between cervical squamous cell carcinoma (SCC) and adenocarcinoma (ADC) samples from our study (x-axis) and from the cancer genome atlas (TCGA) consortium (y-axis). Quadrant II shows 126 genes upregulated in SCC, while 166 genes downregulated in SCC samples in both cohorts (Quadrant III). FC: fold change (log_2); False discovery rate (FDR) < 0.01 was defined as the limit of significance.
