# Supplementary material for: Dysregulation of Transcription Factor Networks Unveils Different Pathways in Human Papillomavirus 16-Positive Squamous Cell Carcinoma and Adenocarcinoma of the Uterine Cervix
Source: Front Oncol. 2021 May 19;11:626187. doi: 10.3389/fonc.2021.626187 (PMC8170088; doi:10.3389/fonc.2021.626187)
Supplement: Supplementary file 4 [file Image_4.pdf]

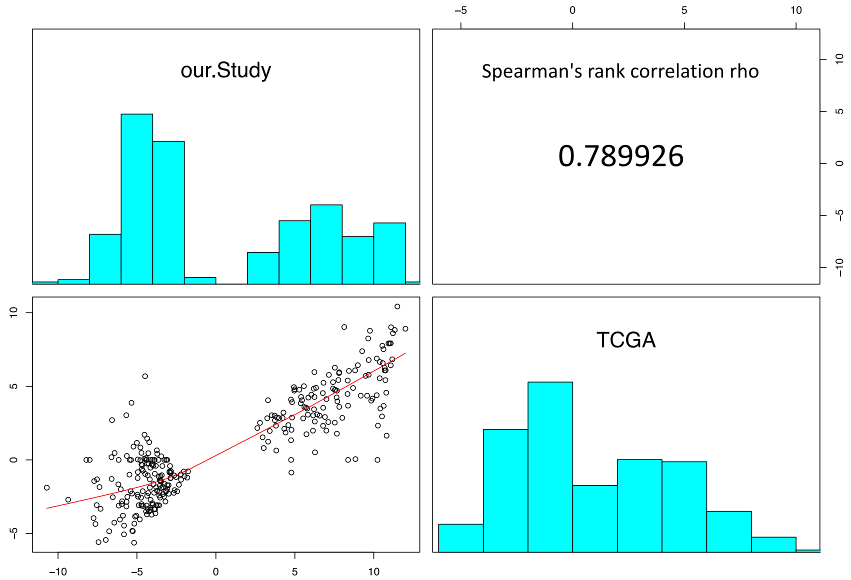

Supplementary Figure 4. Spearman's rank correlation coefficient plot shows the expression signature of differentially genes expression profiles between cervical squamous cell carcinoma (SCC) and adenocarcinoma (ADC) samples from our study and from the cancer genome atlas (TCGA) consortium showed the datasets present the same tendency. FC: fold change (log_2) S = 871690, *p*-value < $2.2e^{-16}$.
