# Supplementary material for: Dysregulation of Transcription Factor Networks Unveils Different Pathways in Human Papillomavirus 16-Positive Squamous Cell Carcinoma and Adenocarcinoma of the Uterine Cervix
Source: Front Oncol. 2021 May 19;11:626187. doi: 10.3389/fonc.2021.626187 (PMC8170088; doi:10.3389/fonc.2021.626187)
Supplement: Supplementary file 5 [file Image_5.pdf]

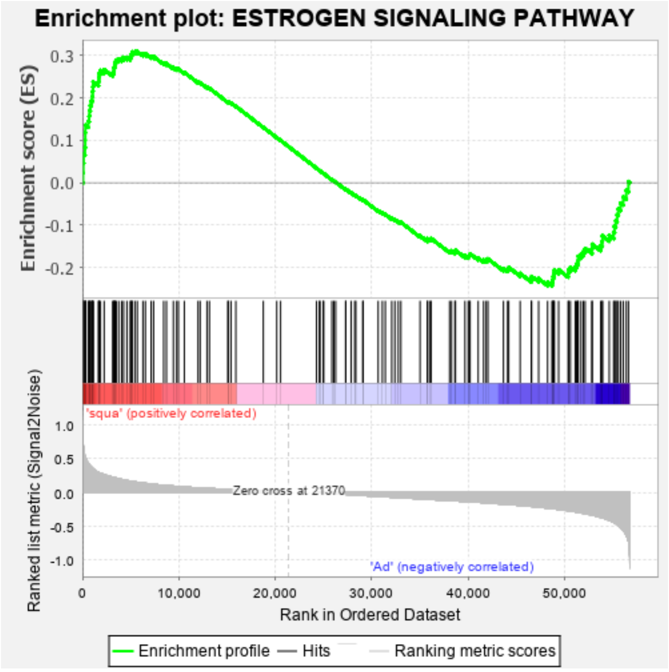


**Supplementary Figure 5.** Gene set enrichment analysis (GSEA) of cervical squamous cell carcinomas (SCC) samples from our study. Genes enriched in cervical SCC that participate in estrogen signaling pathway were identified by GSEA software [12].
