# Supplementary material for: Dysregulation of Transcription Factor Networks Unveils Different Pathways in Human Papillomavirus 16-Positive Squamous Cell Carcinoma and Adenocarcinoma of the Uterine Cervix
Source: Front Oncol. 2021 May 19;11:626187. doi: 10.3389/fonc.2021.626187 (PMC8170088; doi:10.3389/fonc.2021.626187)
Supplement: Supplementary file 6 [file Image_6.pdf]

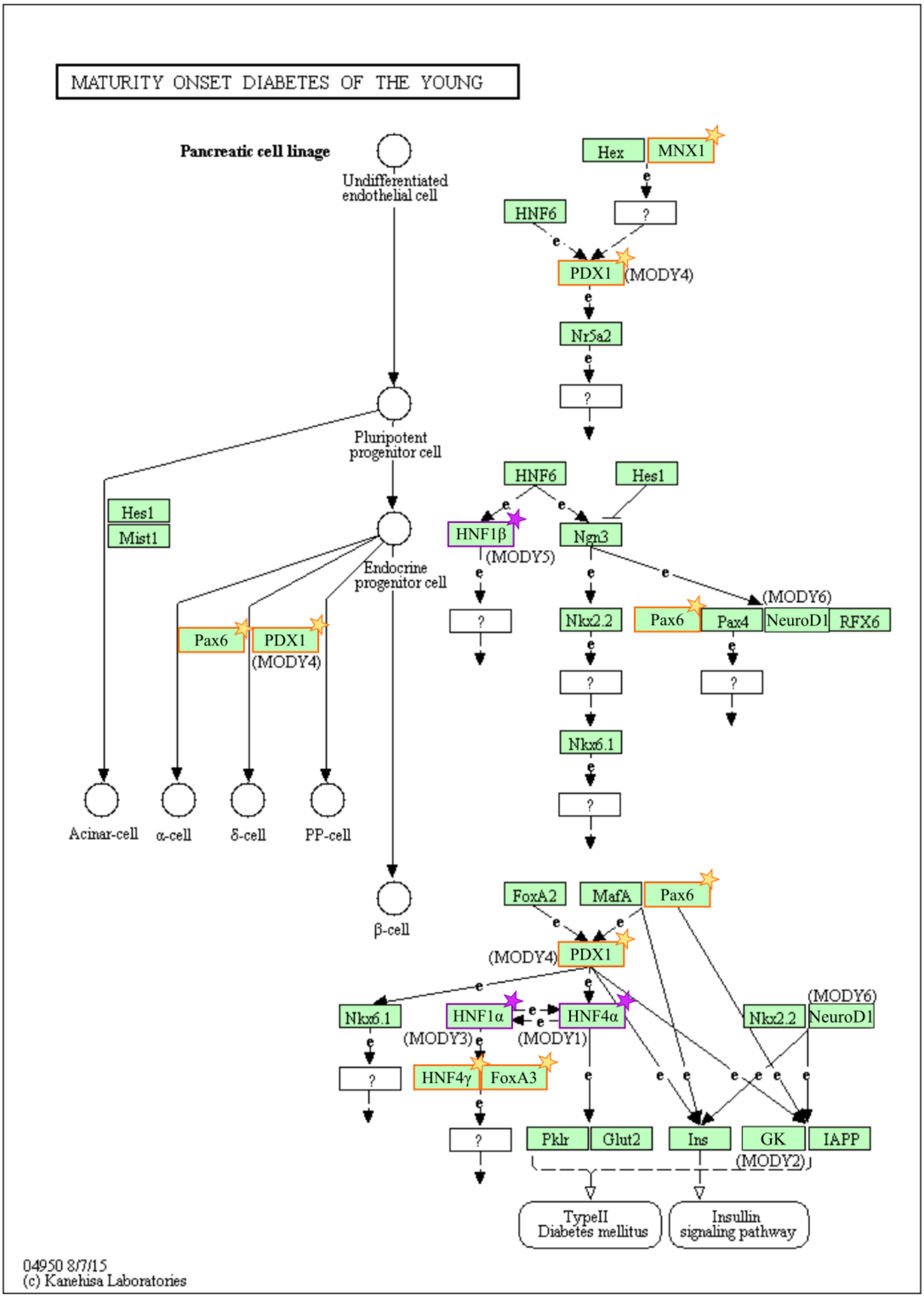


**Supplementary Figure 6.** Kyoto Encyclopedia of Genes and Genomes (KEGG) pathway of genes upregulated in cervical adenocarcinoma (ADC) in our samples that participates of maturity onset diabetes of the young (MODY) pathway. Purple box and stars are genes exclusively expressed in ADC (*HNF1A*, *HNF1B* and *HNF4A*) as compared to cervical SCC and non-CC [10]. Yellow box and stars are genes that are upregulated in ADC samples as compared to cervical SCC in our samples (*PDX1*, *HNF4G*, *FOXA3*) or as compared to non-CC (*PAX6* and *MNX1*). (Table S6, S8).
