# Supplementary material for: Dysregulation of Transcription Factor Networks Unveils Different Pathways in Human Papillomavirus 16-Positive Squamous Cell Carcinoma and Adenocarcinoma of the Uterine Cervix
Source: Front Oncol. 2021 May 19;11:626187. doi: 10.3389/fonc.2021.626187 (PMC8170088; doi:10.3389/fonc.2021.626187)
Supplement: Supplementary file 17 [file Table_11.docx]

| Logistic regression Performance | |
| --- | --- |
| ***AUC ROC (CI95%)*** | 0.898 (0.808; 0.987) |
| ***threshold closest to the top-left AUC ROC*** |  |
| *estimated risk threshold* | 0.077 |
| *TPR (Sensitivity)* | 0.871 (27/31) |
| *TNR (Specificity)* | 0.916 (228/249) |
| *PPV* | 0.563 (27/48) |
| *PNV* | 0.983 (228/232) |
| ***threshold discerning 10% highest risk group*** |  |
| *estimated risk threshold* | 0.208 |
| *TPR (Sensitivity)* | 0.677 (21/31) |
| *TNR (Specificity)* | 0.972 (242/249) |
| *PPV* | 0.750 (21/29) |
| *PNV* | 0.960 (242/252) |

**Supplementary Table 11.** Logistic regression performance for the classifier

*TPR: true positive rate; TNR: true negative rate;

PPV: predictive positive value; PNV: predictive negative value
